# Supplementary material for: Achieving large dynamic range control of gene expression with a compact RNA transcription–translation regulator
Source: Nucleic Acids Res. 2017 Apr 6;45(9):5614–24. doi: 10.1093/nar/gkx215 (PMC5435934; doi:10.1093/nar/gkx215)
Supplement: Supplementary Data [file gkx215_Supp.pdf]

Supporting Information for

Achieving large dynamic range control of gene expression with a compact RNA transcription-translation regulator

Alexandra M. Westbrook[1] and Julius B. Lucks[2,*]

[1] Robert F. Smith School of Chemical and Biomolecular Engineering, Cornell University, Ithaca, NY, 14853, USA
[2] Department of Chemical and Biological Engineering, Northwestern University, Evanston, IL, 60208, USA

* To whom correspondence should be addressed. Tel: 1.847.467.2943; Fax: 1.847.491.3728; Email: jblucks@northwestern.edu

	Description
Table S1	Important DNA sequences
Table S2	Plasmids used in this study
Figure S1	Plasmid architectures used in this study
Note S1	Supplementary Materials and Methods
Figure S2	Day 1, 2, ad 3 of repressor cascade
Figure S3	Repressor cascade from glycerol stock
Figure S4	RepC Knockout
Figure S5	qRT-PCR
Figure S6	Transcriptional activator mechanism
Figure S7	In vivo expression data used to calculate orthogonality matrices
Figure S8	Sequence and structure of the pT181 dual control attenuator
Figure S9	Sequence and structure of the pT181 antisense and truncated pT181 antisense
Figure S10	Induction curves
Table S3	Averages and standard deviations for Figure 2A and 2B
Figure S11	Sequence and structure of the pT181 dual control activator
Table S4	Averages and standard deviations for Figure 3B and 3C
Figure S12	Sequence and structure of interaction regions for antisense mutants and fusions
Table S5	Averages and standard deviations for Figure 4B and 4C
Table S6	Standard deviations for orthogonality matrix repression Figure 5B
Table S7	Standard deviations for orthogonality matrix repression Figure 5C
References	

Table S1: Important DNA sequences

Name	Sequence
J23119	TTGACAGCTAGCTCAGTCCTAGGTATAATACTAGT
pLac Promoter	AATTGTGAGCGGATAACAATTGACATTGTGAGCGGATAACAAGATACT
Super folder green fluorescent protein (Ribosome binding site (RBS) - SFGFP)	AGGAGGAAGGATCTATGAGCAAAGGAGAAGAACTTTTCACTGGAGTTGTCCC<br>AATTCTTGTTGAATTAGATGGTGATGTTAATGGGCACAAATTTTCTGTCCGTGG<br>AGAGGGTGAAGGTGATGCTACAAACGGAAAACTCACCCTTAAATTTATTTGCA<br>CTACTGGAAAACTACCTGTTCCGTGGCCAACACTTGTCACTACTCTGACCTAT<br>GGTGTTCAATGCTTTTCCCGTTATCCGGATCACATGAAACGGCATGACTTTTT<br>CAAGAGTGCCATGCCCGAAGGTTATGTACAGGAACGCACTATATCTTTCAAAG<br>ATGACGGGACCTACAAGACGCGTGCTGAAGTCAAGTTTGAAGGTGATACCCT<br>TGTTAATCGTATCGAGTTAAAGGGTATTGATTTTAAAGAAGATGGAAACATTCT<br>TGGACACAAACTCGAGTACAACTTTAACTCACACAATGTATACATCACGGCAG<br>ACAAACAAAAGAATGGAATCAAAGCTAACTTCAAAATTCGCCACAACGTTGAA<br>GATGGTTCCGTTCAACTAGCAGACCATTATCAACAAAATACTCCAATTGGCGA<br>TGGCCCTGTCCTTTTACCAGACAACCATTACCTGTCGACACAATCTGTCCTTT<br>CGAAAGATCCCAACGAAAAGCGTGACCACATGGTCCTTCTTGAGTTTGTAACT<br>GCTGCTGGGATTACACATGGCATGGATGAGCTCTACAAA
TrrnB	GAAGCTTGGGCCCGAACAAAAACTCATCTCAGAAGAGGATCTGAATAGCGCC<br>GTCGACCATCATCATCATCATCATTGAGTTTAAACGGTCTCCAGCTTGGCTGT<br>TTTGGCGGATGAGAGAAGATTTTCAGCCTGATACAGATTAAATCAGAACGCAG<br>AAGCGGTCTGATAAAACAGAATTTGCCTGGCGGCAGTAGCGCGGTGGTCCCA<br>CCTGACCCCATGCCGAACTCAGAAGTGAAACGCCGTAGCGCCGATGGTAGTG<br>TGGGGTCTCCCCATGCGAGAGTAGGGAACTGCCAGGCATCAAATAAAACGAA<br>AGGCTCAGTCGAAAGACTGGGCCTTTCGTTTTATCTGTTGTTTGTCGGTGAAC<br>T
Monomeric Red fluorescent protein (mRFP)	ATGGCAAGTAGCGAAGACGTTATCAAAGAGTTCATGCGTTTCAAAGTTCGTAT<br>GGAAGGTTCCGTTAACGGTCACGAGTTCGAAATCGAAGGTGAAGGTGAAGGT<br>CGTCCGTACGAAGGTACCCAGACCGCTAAACTGAAAGTTACCAAAGGTGGTC<br>CGCTGCCGTTCGCTTGGGACATCCTGTCCCCGCAGTTCCAGTACGGTTCCAA<br>AGCTTACGTTAAACACCCGGCTGACATCCCGGACTACCTGAAACTGTCCTTCC<br>CGGAAGGTTTCAAATGGGAACGTGTTATGAACTTCGAAGACGGTGGTGTTGTT<br>ACCGTTACCCAGGACTCCTCCCTGCAAGACGGTGAGTTCATCTACAAAGTTAA<br>ACTGCGTGGTACCAACTTCCCGTCCGACGGTCCGGTTATGCAGAAAAAAACC<br>ATGGGTTGGGAAGCTTCCACCGAACGTATGTACCCGGAAGACGGTGCTCTGA<br>AAGGTGAAATCAAAATGCGTCTGAAACTGAAAGACGGTGGTCACTACGACGC<br>TGAAGTTAAAACCACCTACATGGCTAAAAAACCGGTTCAGCTGCCGGGTGCTT<br>ACAAAACCGACATCAAACTGGACATCACCTCCCACAACGAAGACTACACCATC<br>GTTGAACAGTACGAACGTGCTGAAGGTCGTCACTCCACCGGTGCTTAATAA
LacI ORF	GGCACGTAAGAGGTTCCAACTTTCACCATAATGAAACATACTAGAGAAAGAGG<br>AGAAATACTAGATGGTGAATGTGAAACCAGTAACGTTATACGATGTCGCAGAG<br>TATGCCGGTGTCTCTTATCAGACCGTTTCCCGCGTGGTGAACCAGGCCAGCC<br>ACGTTTCTGCGAAAACGCGGGAAAAAGTGGAAGCGGCGATGGCGGAGCTGA<br>ATTACATTCCCAACCGCGTGGCACAACAACTGGCGGGCAAACAGTCGTTGCT<br>GATTGGCGTTGCCACCTCCAGTCTGGCCCTGCACGCGCCGTCGCAAATTGTC<br>GCGGCGATTAAATCTCGCGCCGATCAACTGGGTGCCAGCGTGGTGGTGTCG<br>ATGGTAGAACGAAGCGGCGTCGAAGCCTGTAAAGCGGCGGTGCACAATCTTC<br>TCGCGCAACGCGTCAGTGGGCTGATCATTAACTATCCGCTGGATGACCAGGA<br>TGCCATTGCTGTGGAAGCTGCCTGCACTAATGTTCCGGCGTTATTTCTTGATG<br>TCTCTGACCAGACACCCATCAACAGTATTATTTTCTCCCATGAAGACGGTACG<br>CGACTGGGCGTGGAGCATCTGGTCGCATTGGGTCACCAGCAAATCGCGCTG<br>TTAGCGGGCCCATTAAGTTCTGTCTCGGCGCGTCTGCGTCTGGCTGGCTGGC<br>ATAAATATCTCACTCGCAATCAAATTCAGCCGATAGCGGAACGGGAAGGCGA<br>CTGGAGTGCCATGTCCGGTTTTCAACAAACCATGCAAATGCTGAATGAGGGC<br>ATCGTTCCCACTGCGATGCTGGTTGCCAACGATCAGATGGCGCTGGGCGCAA<br>TGCGCGCCATTACCGAGTCCGGGCTGCGCGTTGGTGCGGATATCTCGGTAG<br>TGGGATACGACGATACCGAAGACAGCTCATGTTATATCCCGCCGTTAACCAC<br>CATCAAACAGGATTTTCGCCTGCTGGGGCAAACCAGCGTGGACCGCTTGCTG<br>CAACTCTCTCAGGGCCAGGCGGTGAAGGGCAATCAGCTGTTGCCCGTCTCAC

	TGGTGAAAAGAAAAACCACCCTGGCGCCCAATACGCAAACCGCCTCTCCCCG<br>CGCGTTGGCCGATTCATTAATGCAGCTGGCACGACAGGTTTCCCGACTGGAA<br>AGCGGGCAGGCTGCAAACGACGAAAACTACGCTTTAGTAGCTTAATAACTCT<br>GATAGTGCTAGTGTAGATCCCTACTAGAGCCAGGCATCAAATAAAACGAAAGG<br>CTCAGTCGAAAGACTGGGCCTTTCGTTTTATCTGTTGTTTGTCGGTGAACGCT<br>CTCTACTAGAGTCACACTGGCTCACCTTCGGGTGGGCCTTTCTGCGTTTATA
Theophylline aptamer-pT181-mutant antisense (aptamer-antisense-sTRSV Ribozyme)	GGTGATACCAGCATCGTCTTGATGCCCTTGGCAGCACCTCTTTGAATGGTGCT<br>GCCCTGCAACTTTGGCGAGGGACAGGGCGACTCCTTTTTATTTCTGTCACCG<br>GATGTGCTTTCCGGTCTGATGAGTCCGTGAGGACGAAACAG
sTRSV Ribozyme	CTGTCACCGGATGTGCTTTCCGGTCTGATGAGTCCGTGAGGACGAAACAG
Double pT181 antisense (BamHI-BglII Scar-sTRSV Ribozyme antisense)x2	GGATCTCTGTCACCGGATGTGCTTTCCGGTCTGATGAGTCCGTGAGGACGAA<br>ACAGGGATCTATACAAGATTATAAAAACAACTCAGTGTTTTTTTCTTTGAATGA<br>TGTCGTTCACAAACTTTGGTCAGGGCGTGAGCGACTCCTTTTTATTTGGATCT<br>CTGTCACCGGATGTGCTTTCCGGTCTGATGAGTCCGTGAGGACGAAACAGGG<br>ATCCTAACTCGAGATACAAGATTATAAAAACAACTCAGTGTTTTTTTCTTTGAAT<br>GATGTCGTTCACAAACTTTGGTCAGGGCGTGAGCGACTCCTTTTTATTTGGAT<br>CT
pT181 repressor (sense target – *repC* 96nt fragment)	AACAAAATAAAAAGGAGTCGCTCACGCCCTGACCAAAGTTTGTGAACGACATC<br>ATTCAAAGAAAAAAACACTGAGTTGTTTTTATAATCTTGTATATTTAGATATTAA<br>ACGATATTTAAATATACATAAAGATATATATTTGGGTGAGCGATTCCTTAAACG<br>AAATTGAGATTAAGGAGTCGCTCTTTTTTATGTATAAAAACAATCATGCAAATC<br>ATTCAAATCATTTGGAAAATCACGATTTAGACAATTTTTCTAAAACCGGCTACT<br>CTAATAGCCGGTTGTAAGGATCT
pT181 repressor with WT terminator (sense target – *repC* 96nt fragment)	AACAAAATAAAAAGGAGTCGCTCACGCCCTGACCAAAGTTTGTGAACGACATC<br>ATTCAAAGAAAAAAACACTGAGTTGTTTTTATAATCTTGTATATTTAGATATTAA<br>ACGATATTTAAATATACATAAAGATATATATTTGGGTGAGCGATTCCTTAAACG<br>AAATTGAGATTAAGGAGTCGATTTTTTATGTATAAAAACAATCATGCAAATCATT<br>CAAATCATTTGGAAAATCACGATTTAGACAATTTTTCTAAAACCGGCTACTCTA<br>ATAGCCGGTTGTAAGGATCT
pT181-mutant 1 repressor (sense target – *repC* 96nt fragment)	AACAAAATAAAAAGGAGTCGCTCTGTCCCTCGCCAAAGTTGCAGAACGACATC<br>ATTCAAAGAAAAAAACACTGAGTTGTTTTTATAATCTTGTATATTTAGATATTAA<br>ACGATATTTAAATATACATAAAGATATATATTTGGGTGAGCGATTCCTTAAACG<br>AAATTGAGATTAAGGAGTCGCTCTTTTTTATGTATAAAAACAATCATGCAAATC<br>ATTCAAATCATTTGGAAAATCACGATTTAGACAATTTTTCTAAAACCGGCTACT<br>CTAATAGCCGGTTGTAAGGATCT
pT181-mutant 2 repressor (sense target – *repC* 96nt fragment)	AACAAAATAAAAAGGAGTCGCTCGTACCCTCTGCAAAGTTAACGAACGACATC<br>ATTCAAAGAAAAAAACACTGAGTTGTTTTTATAATCTTGTATATTTAGATATTAA<br>ACGATATTTAAATATACATAAAGATATATATTTGGGTGAGCGATTCCTTAAACG<br>AAATTGAGATTAAGGAGTCGCTCTTTTTTATGTATAAAAACAATCATGCAAATC<br>ATTCAAATCATTTGGAAAATCACGATTTAGACAATTTTTCTAAAACCGGCTACT<br>CTAATAGCCGGTTGTAAGGATCT
Fusion 3 repressor (sense target – *repC* 96nt fragment)	AACAAAATAAAAAGGAGTCGCTCACGCCTCGAACTTGGCGGAACGCAGTGTG<br>AACGACATCATTCAAAGAAAAAAACACTGAGTTGTTTTTATAATCTTGTATATTT<br>AGATATTAAACGATATTTAAATATACATAAAGATATATATTTGGGTGAGCGATT<br>CCTTAAACGAAATTGAGATTAAGGAGTCGCTCTTTTTTATGTATAAAAACAATC<br>ATGCAAATCATTCAAATCATTTGGAAAATCACGATTTAGACAATTTTTCTAAAAC<br>CGGCTACTCTAATAGCCGGTTGTAAGGATCT
Fusion 4 repressor (sense target – *repC* 96nt fragment)	AACAAAATAAAAAGGAGTCGCTCACGTTCAACTTTGGCGAGTACGATGTGAAC<br>GACATCATTCAAAGAAAAAAACACTGAGTTGTTTTTATAATCTTGTATATTTAGA<br>TATTAAACGATATTTAAATATACATAAAGATATATATTTGGGTGAGCGATTCCTT<br>AAACGAAATTGAGATTAAGGAGTCGCTCTTTTTTATGTATAAAAACAATCATGC<br>AAATCATTCAAATCATTTGGAAAATCACGATTTAGACAATTTTTCTAAAACCGG

	CTACTCTAATAGCCGGTTGTAAGGATCT
pT181 activator (sense target – *repC* 96nt fragment)	TTGGGTGAGCGATTCCTTAAACGAAATTGAGATTAAGGAGTCGCTCTTTTTTTT<br>TTATGTATAAAAACAATCATGCAAATCATTCAAATCATTTGGAAAATCACGATTT<br>AGACAATTTTTCTAAAACCGGCTACTCTAATAGCCGGTTGTAAGGATCT
pT181 dual control activator (sense target – *repC* 12nt fragment)	TTGGGTGAGCGATTCCTTAAACGAAATTGAGATTAAGGAGTCGATTTTTTATGT<br>ATAAAAAC
pT181 dual control repressor with WT terminator (sense target – *repC* 12nt fragment)	AACAAAATAAAAAGGAGTCGCTCACGCCCTGACCAAAGTTTGTGAACGACATC<br>ATTCAAAGAAAAAAACACTGAGTTGTTTTTATAATCTTGTATATTTAGATATTAA<br>ACGATATTTAAATATACATAAAGATATATATTTGGGTGAGCGATTCCTTAAACG<br>AAATTGAGATTAAGGAGTCGATTTTTTATGTATAAAAAC
pT181 dual control repressor (sense target – *repC* 12nt fragment)	AACAAAATAAAAAGGAGTCGCTCACGCCCTGACCAAAGTTTGTGAACGACATC<br>ATTCAAAGAAAAAAACACTGAGTTGTTTTTATAATCTTGTATATTTAGATATTAA<br>ACGATATTTAAATATACATAAAGATATATATTTGGGTGAGCGATTCCTTAAACG<br>AAATTGAGATTAAGGAGTCGCTCTTTTTTATGTATAAAAAC
pT181 mutant 1 dual control repressor (sense target – *repC* 12nt fragment)	AACAAAATAAAAAGGAGTCGCTCTGTCCCTCGCCAAAGTTGCAGAACGACATC<br>ATTCAAAGAAAAAAACACTGAGTTGTTTTTATAATCTTGTATATTTAGATATTAA<br>ACGATATTTAAATATACATAAAGATATATATTTGGGTGAGCGATTCCTTAAACG<br>AAATTGAGATTAAGGAGTCGATTTTTTATGTATAAAAAC
pT181 mutant 2 dual control repressor (sense target – *repC* 12nt fragment)	AACAAAATAAAAAGGAGTCGCTCGTACCCTCTGCAAAGTTAACGAACGACATC<br>ATTCAAAGAAAAAAACACTGAGTTGTTTTTATAATCTTGTATATTTAGATATTAA<br>ACGATATTTAAATATACATAAAGATATATATTTGGGTGAGCGATTCCTTAAACG<br>AAATTGAGATTAAGGAGTCGATTTTTTATGTATAAAAAC
Fusion 3 dual control repressor (sense target – *repC* 12nt fragment)	AACAAAATAAAAAGGAGTCGCTCACGCCTCGAACTTGGCGGAACGCAGTGTG<br>AACGACATCATTCAAAGAAAAAAACACTGAGTTGTTTTTATAATCTTGTATATTT<br>AGATATTAAACGATATTTAAATATACATAAAGATATATATTTGGGTGAGCGATT<br>CCTTAAACGAAATTGAGATTAAGGAGTCGATTTTTTATGTATAAAAAC
Fusion 4 dual control repressor (sense target – *repC* 12nt fragment)	AACAAAATAAAAAGGAGTCGCTCACGTTCAACTTTGGCGAGTACGATGTGAAC<br>GACATCATTCAAAGAAAAAAACACTGAGTTGTTTTTATAATCTTGTATATTTAGA<br>TATTAAACGATATTTAAATATACATAAAGATATATATTTGGGTGAGCGATTCCTT<br>AAACGAAATTGAGATTAAGGAGTCGATTTTTTATGTATAAAAAC
pT181 antisense	ATACAAGATTATAAAAACAACTCAGTGTTTTTTTCTTTGAATGATGTCGTTCACA<br>AACTTTGGTCAGGGCGTGAGCGACTCCTTTTTATTTGGATCT
pT181 mutant 1 antisense	ATACAAGATTATAAAAACAACTCAGTGTTTTTTTCTTTGAATGATGTCGTTCTGC<br>AACTTTGGCGAGGGACAGAGCGACTCCTTTTTATTTGGATCT
pT181 mutant 2 antisense	ATACAAGATTATAAAAACAACTCAGTGTTTTTTTCTTTGAATGATGTCGTTCGTT<br>AACTTTGCAGAGGGTACGAGCGACTCCTTTTTATTTGGATCT

Fusion 4 antisense	ATACAAGATTATAAAAACAACTCAGTGTTTTTTTCTTTGAATGATGTCGTTCACATCGTACTCGCCAAAGTTGAACGTGAGCGACTCCTTTTTATTTGGATCT
Fusion 3 antisense	ATACAAGATTATAAAAACAACTCAGTGTTTTTTTCTTTGAATGATGTCGTTCACACTGCGTTCCGCCAAGTTCGAGGCGTGAGCGACTCCTTTTTATTTGGATCT
pT181 activator antisense	AACAAAATAAAGCAATAAGGAATCGCTCACCCAAAGGATCT
pT181 truncated antisense	TGAATGATGTCGTTCACAAACTTTGGTCAGGGCGTGAGCGACTCCTTTTTGGATCT
Fusion 3 truncated antisense	TGAATGATGTCGTTCACACTGCGTTCCGCCAAGTTCGAGGCGTGAGCGACTCCTTTTTGGATCT
pT181 mutant 1 truncated antisense	TGAATGATGTCGTTCTGCAACTTTGGCGAGGGACAGAGCGACTCCTTTTTGGATCT
pT181 mutant 2 truncated antisense	TGAATGATGTCGTTCGTTAACTTTGCAGAGGGTACGAGCGACTCCTTTTTGGATCT
Fusion 4 truncated antisense	TGAATGATGTCGTTCACATCGTACTCGCCAAAGTTGAACGTGAGCGACTCCTTTTTGGATCT

Table S2 – Plasmids used in this study. Sequences in the plasmid architecture can be found in Table S1.

Plasmid #	Plasmid architecture	Name	Figure	Figure S1 map
JBL001	TrrnB – CmR – p15A origin	CmR/p15A control	2-6, S2-5, S7, S10	B
JBL002	J23119 – TrrnB – ColE1 origin – AmpR	No antisense control	2-6, S2-5, S7, S10	A
JBL003	J23119 – pT181 sense target – *repC*(1-96) – RBS – SFGFP – TrrnB – CmR – p15A origin	pT181 repressor WT term	2	D
JBL004	J23119 – pT181 antisense – TrrnB – ColE1 origin – AmpR	pT181 antisense	2, 4, 5, S4, S5, S7	C
JBL006	J23119 – pT181 sense target mutant terminator – *repC*(1-96) – RBS – SFGFP – TrrnB – CmR – p15A origin	pT181 repressor mut term	2, 4, S5, S10	D
JBL007	J23119 – pT181 mutant 1 sense target mutant terminator – *repC*(1-96) – RBS – SFGFP – TrrnB – CmR – p15A origin	pT181 mutant 1 repressor	4	D
JBL008	J23119 – pT181 mutant 1 antisense – TrrnB – ColE1 origin – AmpR	pT181 mutant 1 antisense	4, 5, S7	C
JBL009	J23119 – pT181 mutant 2 sense target mutant terminator – *repC*(1-96) – RBS – SFGFP – TrrnB – CmR – p15A origin	pT181 mutant 2 repressor	4	D
JBL010	J23119 – pT181 mutant 2 antisense – TrrnB – ColE1 origin – AmpR	pT181 mutant 2 antisense	4, 5, S7	C
JBL021	LacI ORF – pLac – pT181 antisense – TrrnB – ColE1 origin – AmpR	IPTG inducible antisense	S10	E
JBL1033	J23119 – Fusion 4 antisense – TrrnB – ColE1 origin – AmpR	Fusion 4 antisense	4, 5, S7	C
JBL1035	J23119 – Fusion 3 antisense – TrrnB – ColE1 origin – AmpR	Fusion 3 antisense	4, 5, S7	C
JBL1039	J23119 – Fusion 3 sense target mutant terminator – *repC*(1-96) – RBS – SFGFP – TrrnB – CmR – p15A origin	Fusion 3 repressor	4	D

JBL1126	J23119 – Fusion 4 sense target mutant terminator – *repC*(1-96) – RBS – SFGFP – TrrnB – CmR – p15A origin	Fusion 4 repressor	4	D
JBL1843	J23119 – theophylline aptamer-pT181 mutant 1 antisense – sTRSV ribozyme – TrrnB – ColE1 origin – AmpR	Aptamer pT181 mutant 1 antisense, aptamer-AS-2	6, S2, S3	G
JBL1844	J23119 – pT181 mutant 1 sense target mutant terminator – *repC*(1-96) – (sTRSV ribozyme – pT181 antisense)x2 – TrrnB – CmR – p15A origin	Cascade L2 on p15A/CmR backbone	6, S2, S3	H
JBL1855	J23119 – pT181 sense target – *repC*(1-96) – RBS – SFGFP – TrrnB – pSC101 origin – KanR	pT181 repressor	6, S2, S3	I
JBL1856	TrrnB – pSC101 origin – KanR	pSC101/KanR control	6, S2, S3	J
JBL2071	J23119 – pT181 activator – RBS – SFGFP – TrrnB – CmR – p15A origin	pT181 activator	3	D
JBL2128	J23119 – pT181 activator antisense – TrrnB – ColE1 origin – AmpR	pT181 activator antisense	3	C
JBL2412	J23119 – pT181 dual control sense target – *repC*(1-12) – SFGFP – TrrnB – CmR – p15A origin	pT181 DC repressor WT term	2, 4, 5, S4, S5, S7, S10	D
JBL2413	J23119 – pT181 dual control sense target mutant terminator – *repC*(1-12) – SFGFP – TrrnB – CmR – p15A origin	pT181 DC repressor	2	D
JBL2415	J23119 – pT181 dual control mutant 1 sense target – *repC*(1-12) – SFGFP – TrrnB – CmR – p15A origin	pT181 DC mutant 1 repressor	4, 5, S7	D
JBL2421	J23119 – pT181 dual control activator sense target – *repC*(1-12) – SFGFP – TrrnB – CmR – p15A origin	pT181 DC activator	4, 5, S7	D
JBL2427	J23119 – Fusion 3 dual control sense target – *repC*(1-12) – SFGFP – TrrnB – CmR – p15A origin	Fusion 3 DC repressor	4, 5, S7	D
JBL2432	J23119 – pT181 dual control mutant 2 sense target – *repC*(1-12) – SFGFP – TrrnB – CmR – p15A origin	pT181 DC mutant 2 repressor	4, 5, S7	D
JBL2434	J23119 – Fusion 4 dual control sense target – *repC*(1-12) – SFGFP – TrrnB – CmR – p15A origin	Fusion 4 DC repressor	4, 5, S7	D
JBL2463	J23119 – pT181 dual control sense target – *repC*(1-12) – RFP – RBS – SFGFP – TrrnB – CmR – p15A origin	Dual control RFP/GFP	2	F
JBL2464	J23119 – pT181 truncated antisense – TrrnB – ColE1 origin – AmpR	pT181 truncated antisense	5, S7	C
JBL2469	J23119 – Fusion 3 truncated antisense – TrrnB – ColE1 origin – AmpR	Fusion 3 truncated antisense	5, S7	C
JBL2489	J23119 – pT181 mutant 1 truncated antisense – TrrnB – ColE1 origin – AmpR	pT181 mutant 1 truncated antisense	5, S7	C
JBL2490	J23119 – pT181 mutant 2 truncated antisense – TrrnB – ColE1 origin – AmpR	pT181 mutant 2 truncated antisense	5, S7	C
JBL2491	J23119 – Fusion 4 truncated antisense – TrrnB – ColE1 origin – AmpR	Fusion 4 truncated antisense	5, S7	C
JBL2493	J23119 – pT181 dual control sense target – *repC*(1-12) – SFGFP – TrrnB – pSC101 origin – KanR	pT181 DC WT Term repressor	6, S2, S3	I
JBL2526	J23119 – pT181 dual control sense target – SFGFP – TrrnB – CmR – p15A origin	pT181 DC repressor RepC KO	S4	D

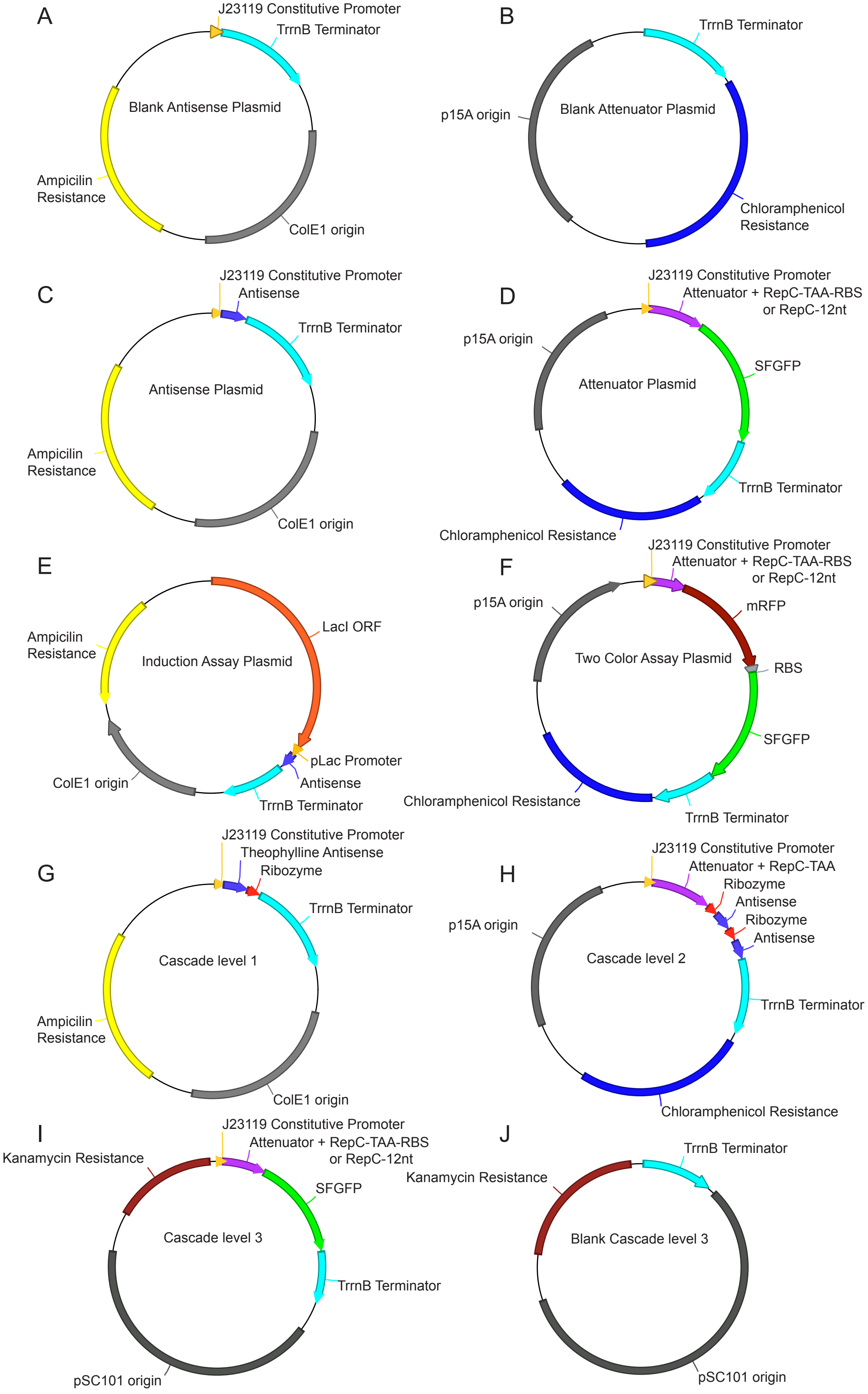
A
J23119 Constitutive Promoter
TrrnB Terminator
Blank Antisense Plasmid
Ampicilin Resistance
ColE1 origin
B
TrrnB Terminator
p15A origin
Blank Attenuator Plasmid
Chloramphenicol Resistance
C
J23119 Constitutive Promoter
Antisense
TrrnB Terminator
Antisense Plasmid
Ampicilin Resistance
ColE1 origin
D
J23119 Constitutive Promoter
Attenuator + RepC-TAA-RBS or RepC-12nt
p15A origin
SFGFP
Attenuator Plasmid
TrrnB Terminator
Chloramphenicol Resistance
E
Ampicilin Resistance
LacI ORF
Induction Assay Plasmid
pLac Promoter
Antisense
ColE1 origin
TrrnB Terminator
F
J23119 Constitutive Promoter
Attenuator + RepC-TAA-RBS or RepC-12nt
p15A origin
mRFP
Two Color Assay Plasmid
RBS
SFGFP
Chloramphenicol Resistance
TrrnB Terminator
G
J23119 Constitutive Promoter
Theophylline Antisense
Ribozyme
TrrnB Terminator
Cascade level 1
Ampicilin Resistance
ColE1 origin
H
J23119 Constitutive Promoter
Attenuator + RepC-TAA
Ribozyme
Antisense
Ribozyme
Antisense
p15A origin
Cascade level 2
TrrnB Terminator
Chloramphenicol Resistance
I
J23119 Constitutive Promoter
Attenuator + RepC-TAA-RBS or RepC-12nt
Kanamycin Resistance
SFGFP
Cascade level 3
TrrnB Terminator
pSC101 origin
J
TrrnB Terminator
Kanamycin Resistance
Blank Cascade level 3
pSC101 origin

Supplementary Figure S1: Plasmid architectures for plasmids used in this study. (A) Antisense plasmid blank control (B) Attenuator plasmid blank control (C) Antisense plasmid architecture (D) Attenuator plasmid architecture (E) Induction assay plasmid architecture (F) Two color assay plasmid architecture (G) Architecture of level 1 (top level) of the cascade (H) Architecture of level 2 (mid level) of the cascade (I) Architecture of level 3 (bottom level) of the cascade (J) Cascade level 3 (bottom level) blank control plasmid. Specific sequences can be found in Table S1.

Supplementary Note S1: Supplementary Materials and Methods.

Total RNA extraction for quantitative PCR. qRT-PCR was performed following the MIQE guidelines (1). Strain, transformation, media, and growth conditions were all the same as for end point experiments described in the Materials in Methods in the main text. Plasmids were transformed, and subsequent colonies were grown overnight as described for *in vivo* bulk fluorescence measurements. For each biological replica, 20 μl of a single overnight culture was added to three wells containing 980 μl (1:50 dilution) of supplemented M9 minimal medium containing the selective antibiotics and grown for 4 h at the same conditions as the overnight cultures. For each plasmid combination, 500 μl of cells were removed from three wells (grown from one colony) and combined into a 1.6-ml tube and pelleted by centrifugation at 13,000 r.p.m. for 1 min. Total RNA extraction was performed using Trizol reagent (Life Technologies) and an ethanol precipitation as described in Chappell et al. (2). The supernatant was removed, and the remaining pellet was resuspended in 750 μl of Trizol reagent (Life Technologies), homogenized by repetitive pipetting, and incubated at room temperature for 5 min. 150 μl of chloroform was added, and the samples were mixed for 15 s and incubated at room temperature for 3 min. Following incubation, the samples were centrifuged for 15 min at 12,000g at 4 °C, and 200 μl of the top aqueous layer was removed. One microliter of glycogen (20 μg/μl; Life Technologies) and 375 μl of isopropanol were added to the aqueous phase, and the sample was incubated at room temperature for 10 min and centrifuged for 15 min at 15,000 r.p.m. at 4 °C. Following centrifugation, the isopropanol was carefully removed from the total RNA/glycogen pellets, washed in 600 μl of chilled 70% ethanol (EtOH) and centrifuged for 2 min at 15,000 r.p.m. at 4 °C. EtOH was removed, and tubes were centrifuged for another 2 min at 15,000 r.p.m. at 4 °C to ensure that all of the ethanol was effectively removed. Pellets were resuspended in 20 μl of RNase free double-distilled water (ddH2O) and stored at -80 °C.

DNase treatment of total RNA for qPCR. The total RNA was treated with Turbo DNase to remove any genomic or plasmid DNA from the RNA extraction (2). Purified total RNA samples were quantified by the Qubit Fluorometer (Life Technologies) and were diluted to a concentration of 30 ng/μl in a total of 10 μl RNase free ddH2O and digested by Turbo DNase (Life Technologies) according to the manufacturer's protocol. After digestion, 150 μl of RNase free ddH2O and 200 μl phenol/chloroform was added, and the sample was vortexed for 10 s and incubated for 3 min at room temperature and centrifuged for 10 min at 15,000 r.p.m. at 4 °C. After centrifugation, 190 μl of the top aqueous layer was carefully removed, 190 μl of chloroform was added, and samples were vortexed for 10 s, incubated for 3 min at room temperature and centrifuged for 10 min at 15,000 r.p.m. at 4 °C. After centrifugation, 170 μl of the top aqueous layer was carefully removed, 170 μl of chloroform was added, and samples were vortexed for 10 s, incubated for 3 min at room temperature and centrifuged for 10 min at 15,000 r.p.m. at 4 °C. After centrifugation, 120 μl of the top aqueous layer was carefully removed and added to 1 μl glycogen, 360 μl of chilled 100% EtOH and 12 μl of 3 M sodium acetate, pH 5. Samples were vortexed for 10 s and stored at −80 °C for 1 h. Samples were then centrifuged for 30 min at 15,000 r.p.m. at 4 °C. Supernatant was removed, and the pellets were washed in 600 μl of chilled 70% EtOH. Samples were then centrifuged for 2 min at 15,000 r.p.m. at 4 °C, and the EtOH was removed. Samples were recentrifuged for 2 min at 15,000 r.p.m. at 4 °C, and residual EtOH was removed, and pellets were air-dried for 10 min, and eluted in 10 μl RNase fee ddH2O and stored at -80 °C.

Normalization of total RNA, reverse transcription and qPCR measurements. To enable comparison between different samples, each DNase treated sample was normalized to contain the same total RNA concentration. Each sample was quantified by Qubit Fluorometer, and the sample was diluted to 1 ng/μl of total RNA in 12 μl RNase free ddH2O. For the reverse transcription, one microliter of this total RNA, 1 μl of 2 μM reverse transcription primer (RT

SFGFP: TTATTTGTAGAGCTCATCCATG), 1 µl of 10 mM of dNTPs (New England BioLabs) and RNase-free ddH2O (up to 6.5 µl) were incubated for 5 min at 65 °C and cooled on ice for 5 min. 0.25 µl of Superscript III reverse transcriptase (Life Technologies), 1 µl of 100 mM Dithiothreitol (DTT), 1 µl first-strand buffer (Life Technologies), 0.5 µl RNaseOUT (Life Technologies) and RNase free H2O up to 3.5 µl were then added, and the solution was incubated at 55 °C for 1 h, 75 °C for 15 min and then stored at −20 °C. qPCR was performed using 5 µl of Maxima SYBR green qPCR master mix (Thermo Scientific), 1 µl of cDNA and 0.5 µl of 2 µM SFGFP qPCR primers (SFGFP.Fwd: CACTGGAGTTGTCCCAATTCT, SFGFP.Rev: TCCGTTTGTAGCATCACCTTC) and RNase-free ddH2O up to 10 µl. A Bio-Rad CFX Connect Real-Time System (Bio-Rad) was used for data collection using the following PCR program: 50 °C for 2 min, 95 °C for 10 min, followed by 30 cycles of 95 °C for 15 s and 60 °C for 1 min. All of the measurements were followed by melting curve analysis. A Hard-Shell 96-well PCR Plate (HSL9641, Bio-Rad) and a Microseal 'B' seal (MSB1001, Bio-Rad) were used for all measurements. Results were analyzed using Bio-Rad CFX Manager (V 3.1, Bio-Rad) by a relative standard curve. For quantification, a six-point standard curve covering a 100,000-fold range of SFGFP DNA concentrations ($R^2 > 0.99$) was run in parallel and used to determine the relative SFGFP cDNA abundance in each sample. It was shown that the SFGFP qPCR primer set had a primer efficiency between 101–104%. All of the cDNA samples were measured in triplicate, and nontemplate controls run in parallel to control for contamination and nonspecific amplification or primer dimers. All NTC samples were quantified after 30 cycles. In addition, qPCR was performed on total RNA samples to confirm limited plasmid DNA contamination of cDNA samples. Control samples were quantified at least 4 cycles after their cDNA samples. Melting curve analysis was performed to confirm that only a single product was amplified. Units are reported as ng/µl x 10^{-7} representing cDNA concentration in the processed samples.

Induction curve assay. Strain, transformation, media, and growth conditions were all the same as for end point experiments described in the Materials and Methods in the main text. Plasmid combinations were transformed into chemically competent E. coli TG1 cells, plated on Difco LB+Agar plates containing selective antibiotics and incubated overnight (approximately 17 hours) at 37°C. Plates were taken out of the incubator and left at room temperature for approximately 7 h. Three colonies were used to separately inoculate 300 µL of LB containing selective antibiotics in a 2 mL 96-well block (Costar 3960), and grown approximately 17 h overnight at 37°C. Four microliters of this overnight culture was then added to 196 µL (1:50 dilution) of supplemented M9 minimal media containing the selective antibiotics and the required amount of IPTG. After 4 h of incubation at 37C, 50 µL of this culture was then transferred to a 96-well plate (Costar 3631) containing 50 µL of phosphate buffered saline (PBS). Fluorescence (485 nm excitation, 520 nm emission) and optical density (OD, 600 nm) were then measured using a Biotek SynergyH1m plate reader.

RepC Knockout Assay. Strain, Transformation, media, and growth conditions were all the same for the end point experiments described in the Materials and Methods in the main text. After the 4 h incubation at 37C in supplemented M9 minimal media, 50 µL of this culture was then transferred to a 96-well plate (Costar 3631) containing 50 µL of phosphate buffered saline (PBS). Fluorescence (485 nm excitation, 520 nm emission) and optical density (OD, 600 nm) were then measured using a Biotek SynergyH1m plate reader.

***In Vivo* bulk fluorescence time course experiments from glycerol stocks,** Strain, transformation, media, and growth conditions were all the same as for end point experiments described in the Materials in Methods in the main text. Transformation plates containing *E. coli* TG1 cells transformed with three cascade plasmids (Supplementary Table S2) were taken out of the incubator and left at room temperature for approximately 7 h. Colonies were picked and incubated at 37C overnight in LB containing selective antibiotics. The following morning, 500µL of culture was mixed with 500µL of 50% glycerol and frozen at -80C. Three colonies were picked from the frozen glycerol stocks and used to inoculate 300 µL of LB containing selective antibiotics in a 2 mL 96-well block (Costar 3960), and grown approximately 17 h overnight at the same conditions as described for an end point experiment (see Materials and Methods in main text). Cultures were diluted 1:50 in separate wells on a new block containing 1 mL of supplemented M9 minimal media containing the selective antibiotics and grown for 4 h at the same conditions as the overnight culture. Then theophylline was added to the theophylline condition to a final concentration of 2mM. Every 30 min for the next 4 h, 50 µL from each of the fresh cultures was

removed from the 96-well block and transferred to a 96-well plate (Costar 3631) containing 50 μL of phosphate buffered saline (PBS). SFGFP fluorescence (FL, 485 nm excitation, 520 nm emission) and optical density (OD, 600 nm) were then measured at each time point using a Biotek Synergy H1m plate reader.

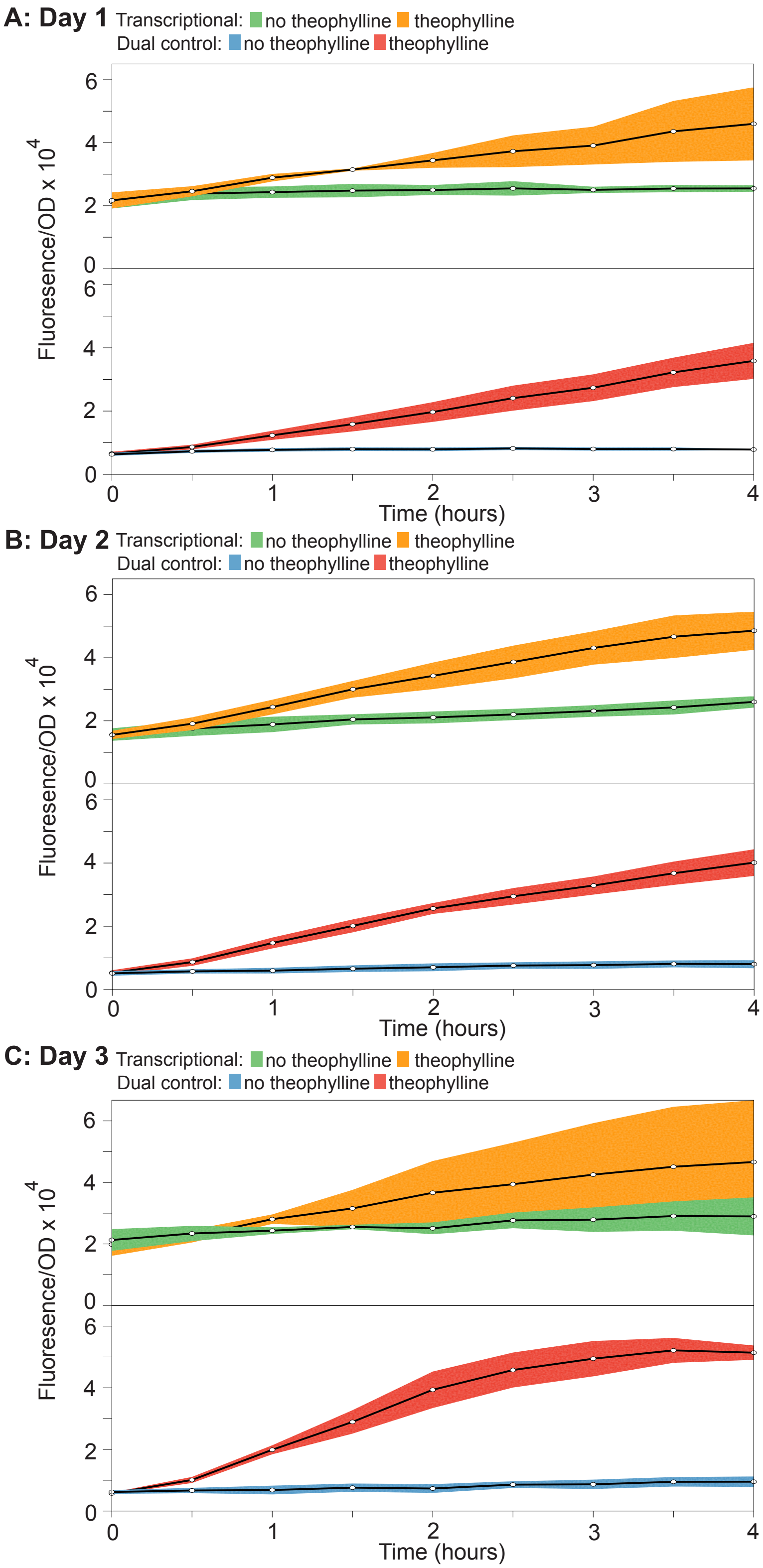

Supplementary Figure S2: Additional data for repressor cascade (Figure 6). Functional data for the transcriptional and dual control repressor cascades over time. All three plasmids were co-transformed into *E. coli* TG1 cells. Theophylline (2mM) is spiked at t=0 hours (orange for transcriptional and red for dual control) causing GFP to be expressed. Bulk fluorescence was measured using a plate reader (see Materials and Methods). The no theophylline condition is shown in green for the transcriptional cascade and blue for dual control. The dual control reduces circuit leak and the background fluorescence. The transformation, culturing, and measurements were done on different days for three days of experiments. The colored region indicates the standard deviation from three biological replicates.

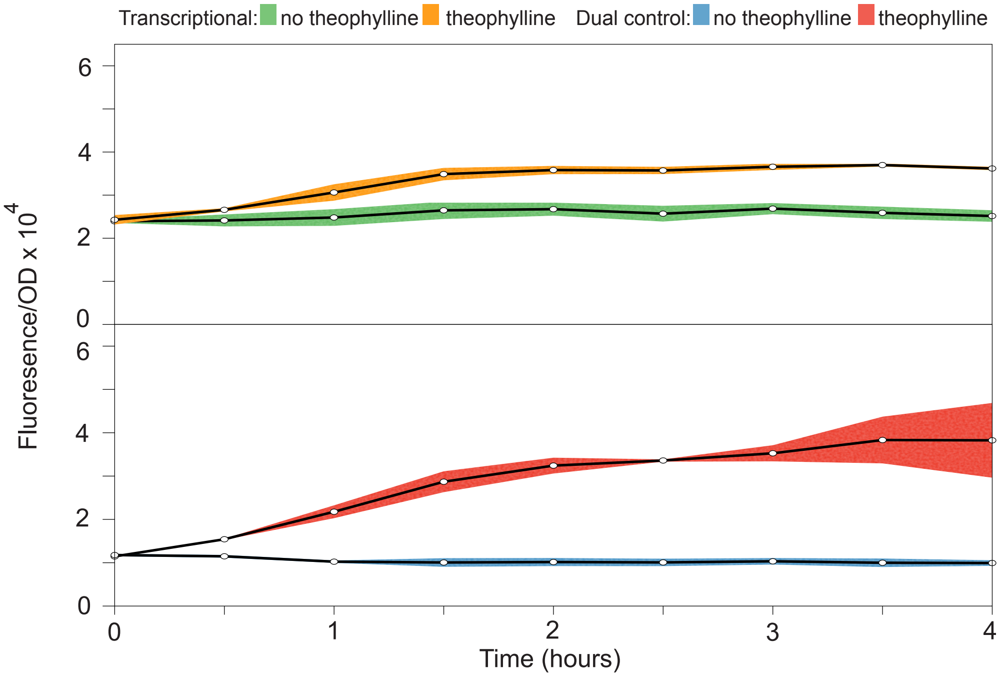

Supplementary Figure S3: Functional data for the transcriptional and dual control repressor cascades over time performed from glycerol stocked strains. Experiment performed as described in Supplementary Note S1. Data was analyzed as described in the Materials and Methods. The no theophylline condition is shown in green for the transcriptional cascade and blue for dual control. The dual control reduces circuit leak and the background fluorescence. The colored region indicates the standard deviation from three biological replicates.

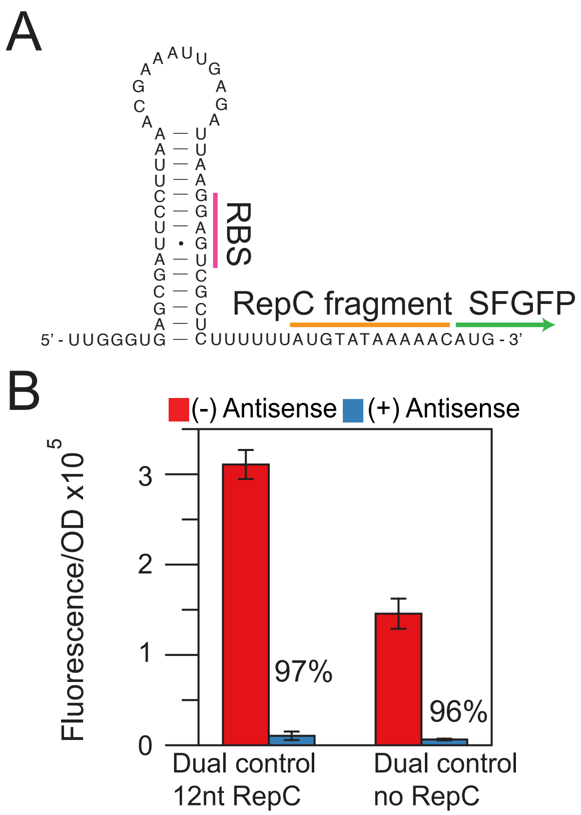

Supplementary Figure S4: Functionality of the 12nt RepC fusion versus complete RepC knockout. (A) Schematic of the pT181 transcriptional terminator with RBS (pink) and dual control 12nt fusion to RepC (orange) expressing SFGFP (green). The dual control with no RepC lacks the orange region. (B) Experiments were performed as described in Supplementary Note S1. Bulk fluorescence data was collected using a plate reader. The 12nt RepC fusion shows similar percent repression (97% +/- 1.5%) as the dual control with no RepC (96% +/-0.8%), but has a much higher ON level. Error bars represent the standard deviation of nine biological replicates.

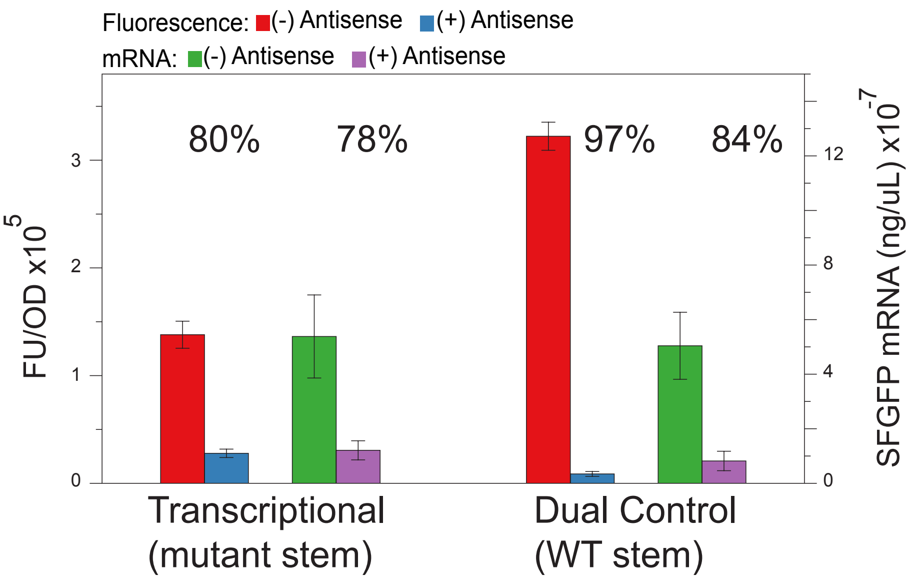

Supplementary Figure S5: Quantitative RT-PCR of a transcriptional and a dual control pT181 attenuator. qRT-PCR quantification was performed as described in Supplementary Note S1. Bulk fluorescence was measured using a plate reader from the same cultures as the RNA was extracted. The fluorescence ON levels (red) were significantly different ($P > 0.05$ for Welch's t-test), but mRNA ON levels (green) were not significantly different. Transcriptional fluorescence repression (80% +/- 3.4%) was similar to mRNA repression (78 +/- 9.1%) while dual control fluorescence repression (97% +/- 0.7%) was much higher than mRNA repression (84% +/- 8.1%). The error bars represent the standard deviation of three technical replicates of three biological replicates.

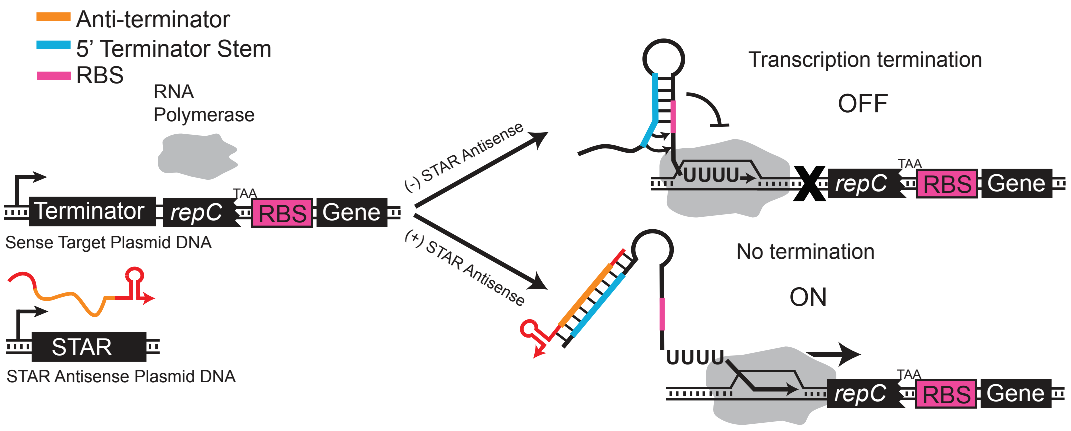

Supplementary Figure S6: Schematic of the pT181 anti-terminator STAR mechanism. The sense target region consists of the pT181 STAR target region from Chappell et al. (2) followed by a 96 nt fragment of the *repC* gene ending in a stop codon, TAA, included as a transcriptional fusion before a ribosome binding site (RBS) and the regulated gene of interest. In the absence of the STAR RNA (red/orange), the terminator forms, preventing downstream transcription by RNA polymerase (grey). Thus in the absence of STAR RNA the mechanism is transcriptionally OFF. The STAR RNA contains an anti-terminator sequence (orange) complementary to the 5' half of the terminator (blue). When present, the STAR RNA binds to the terminator, preventing terminator formation and allowing transcription elongation. Thus in the presence of STAR RNA the mechanism is transcriptionally ON.

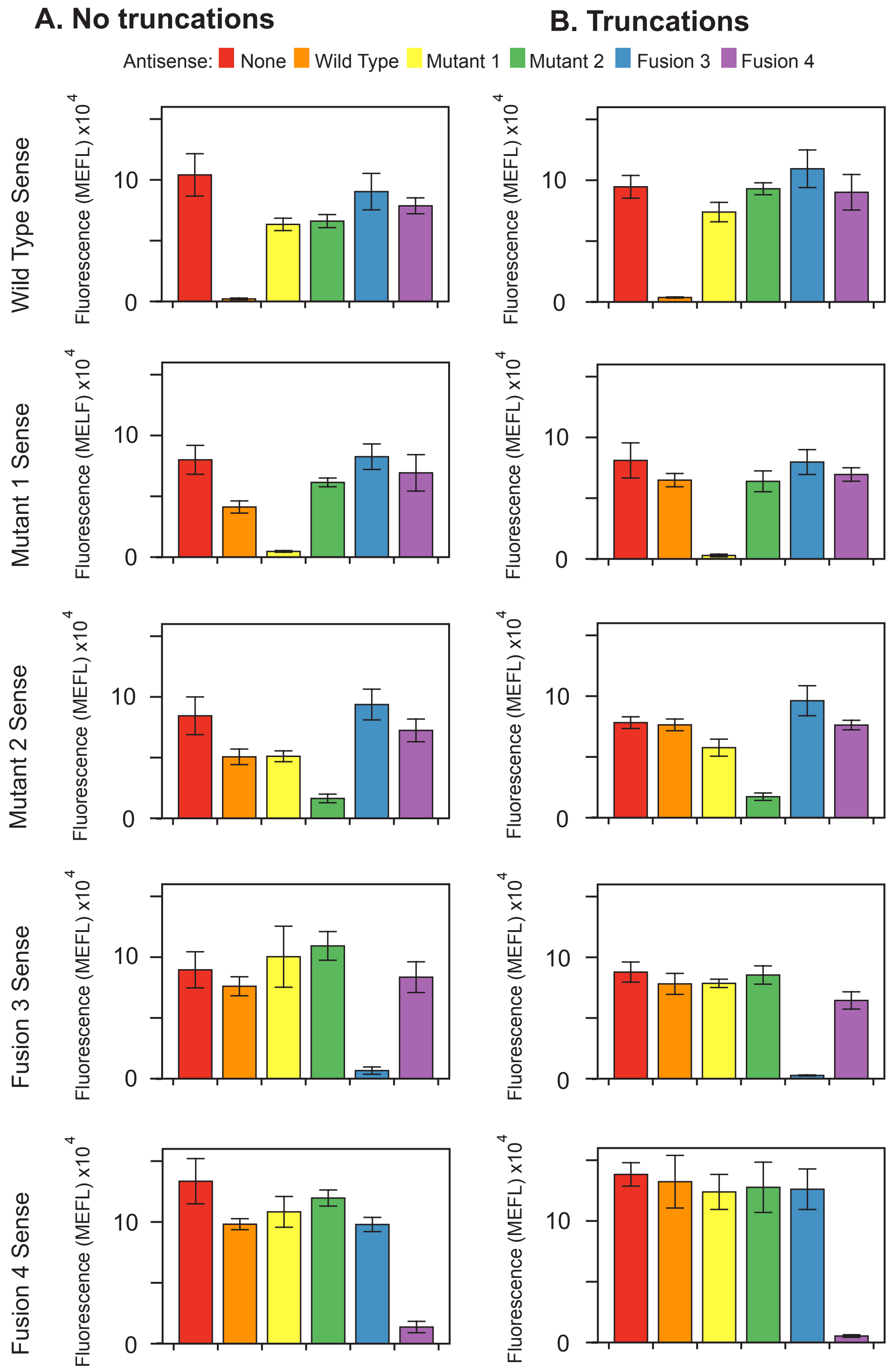

Supplementary Figure S7: In vivo expression data used to calculate orthogonality matrices in Figure 5. Functional characterization of orthogonality matrix cognate and non-cognate pairs. Average fluorescence (MEFL) was collected by flow cytometry of *E. coli* TG1 cells with no antisense (red), wild type antisense (orange), mutant 1 antisense (yellow), mutant 2 antisense (green), fusion 3 antisense (blue), or fusion 4 antisense (purple). Error bars represent standard deviations of at least seven biological replicates.

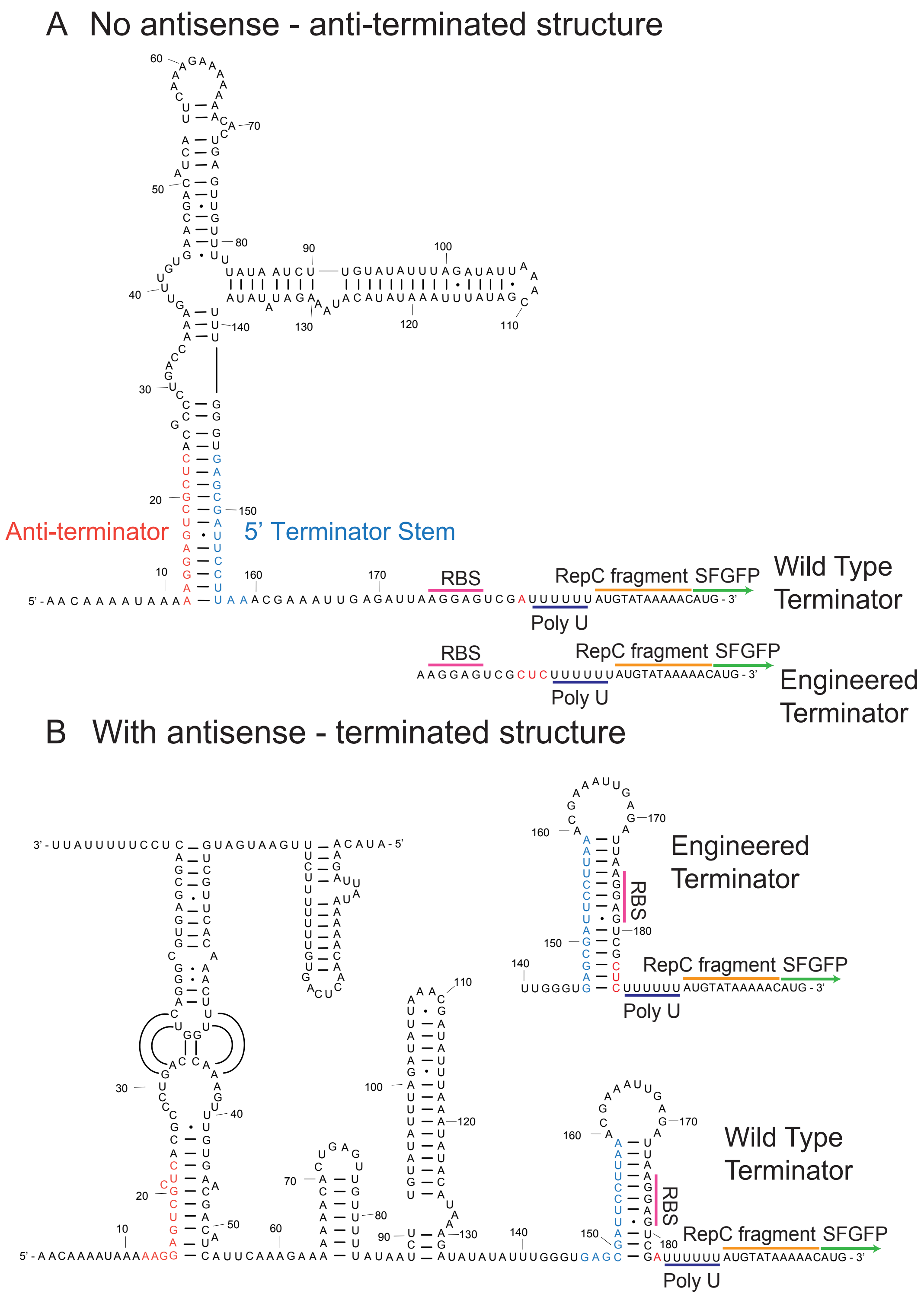

Supplementary Figure S8: Sequences and model structures of the schematic shown in Figure 1B with both the anti-terminated structure (ON) in the absence of antisense (A) and the terminated structure (OFF) in the presence of antisense (B). The anti-terminator region is highlighted in orange and the 5' terminator stem is highlighted in blue. The RBS is shown with a pink line, the Poly U with a blue line, the 12 nt RepC fragment with an orange line, and SFGFP coding sequence with a green line. Sequences for both the wild type and engineered terminators are shown. Structures are from Brantl and Wagner (3).

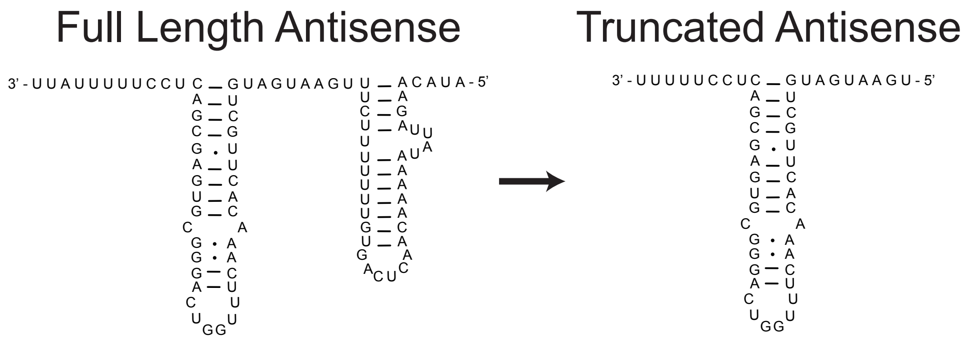

Supplementary Figure S9: Sequence and structure of the full length wild type pT181 antisense and the truncated wild type antisense. In order to create the truncated antisense, the first hairpin from the 5’ side was removed and sequence was truncated from the 5’ stem of the antisense. Structures are from Brantl and Wagner (3).

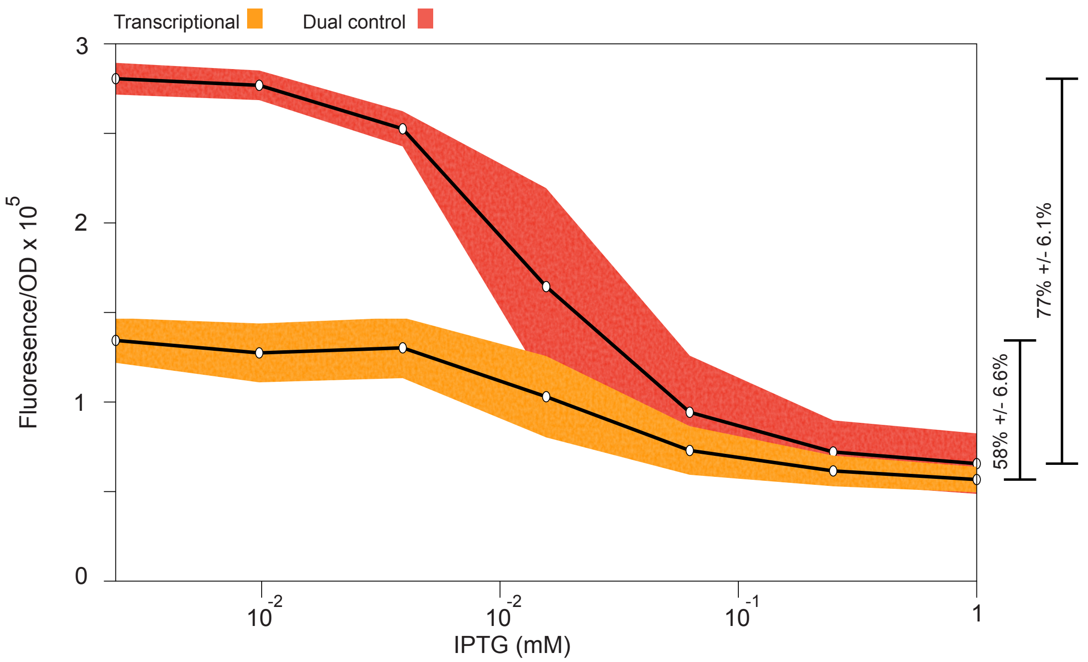

Supplementary Figure S10: Induction curves of transcriptional and dual control attenuators. Antisense expression is under the control of the Lac promoter. Experiment performed as described in Supplementary Note S1. Data was analyzed as described in Materials and Methods. The dual control attenuator (red) shows greater dynamic range (77% +/- 6.1%) in response to IPTG induction than the transcriptional (orange) attenuator (58% +/- 6.6%). The colored region indicates the standard deviation from 3 biological replicates.

Supplementary Table S3: Averages and standard deviations (MEFL) plotted in Figure 2A and 2B. Values have been rounded to the nearest integer for ease of interpretation.

	No antisense (average)	With antisense (average)	No antisense (SD)	With antisense (SD)
Transcriptional WT terminator	40289	14857	7307	1659
Dual control WT terminator	94148	1630	11530	260
Transcriptional Eng terminator	38699	5849	4207	1132
Dual control Eng terminator	27401	671	3644	181

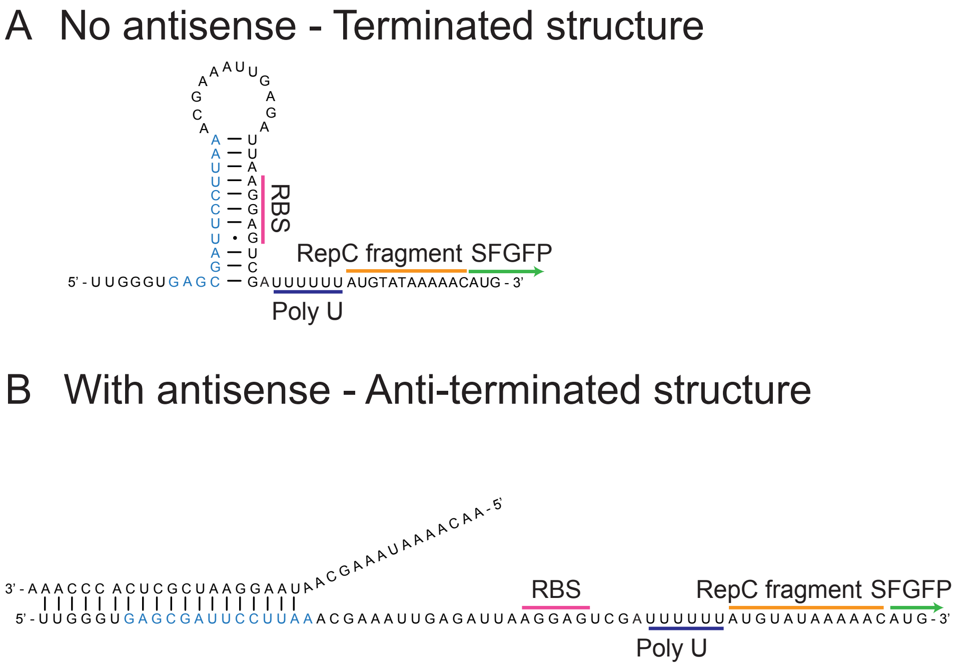

Supplementary Figure S11: Sequences and structures of the schematic shown in Figure 3A with both the terminated structure (OFF) in the absence of antisense (A) and the anti-terminated structure (ON) in the presence of antisense (B). The 5' terminator stem is highlighted in blue. The RBS is shown with a pink line, the Poly U with a blue line, the 12 nt RepC fragment with an orange line, and SFGFP with a green line. Structures are derived from diagrams in Chappell et al. (2).

Supplementary Table S4: Averages and standard deviations (MEFL) plotted in Figure 3B and 3C. Values have been rounded to the nearest integer for ease of interpretation.

	No antisense (average)	With antisense (average)	No antisense (SD)	With antisense (SD)
STAR	3700	37976	695	11837
Dual control STAR	115	106062	16	19872

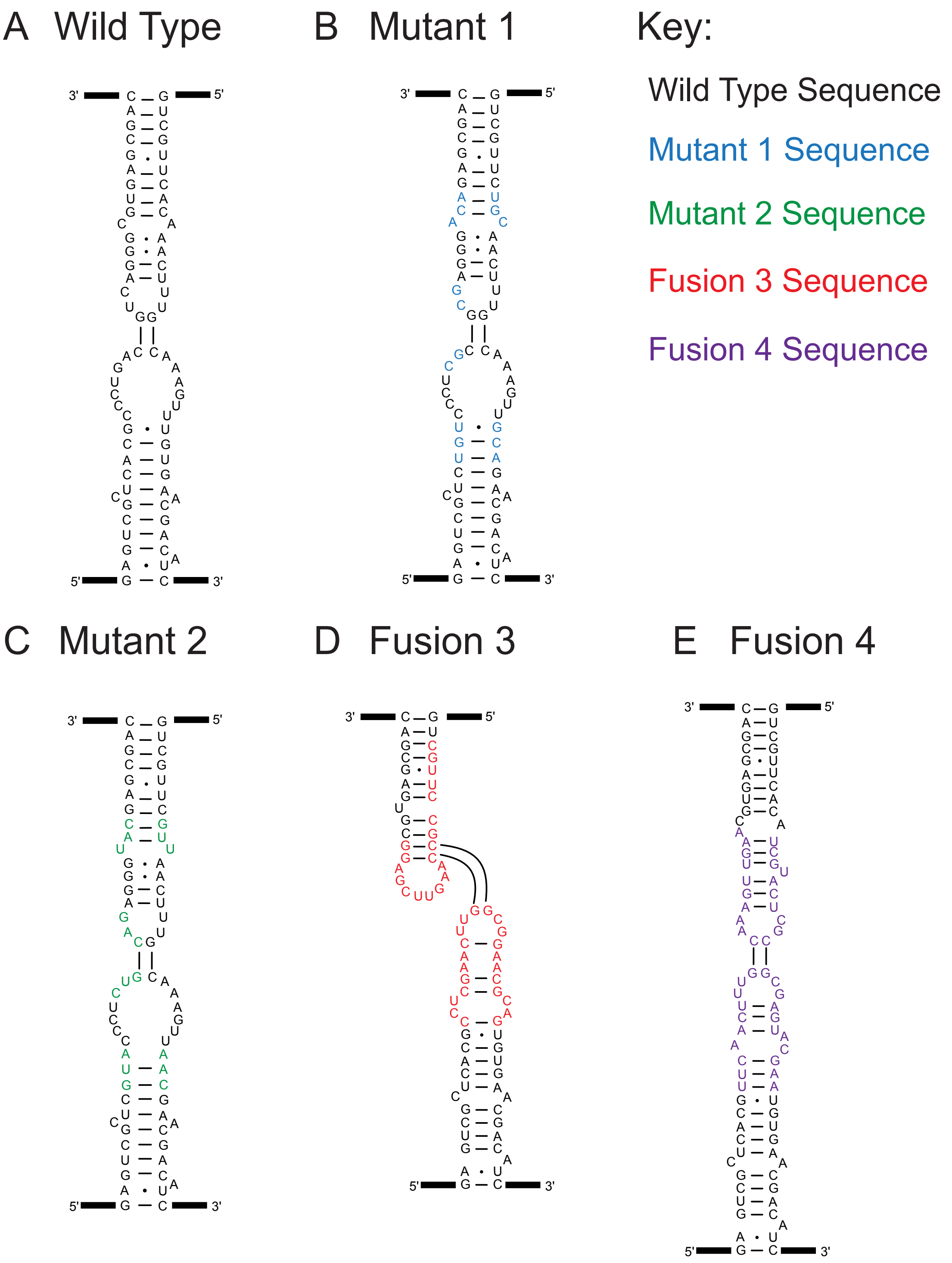

Supplementary Figure S12: Sequences and structures of antisense/attenuator interaction regions in Figure 4A including the wild type (A), mutant 1 (B), mutant 2 (C), fusion 3 (D), and fusion 4 (E). Wild type sequence is shown in black. Mutant 1 sequence is shown in blue. Mutant 2 sequence is shown in green. Fusion 3 sequence is shown in red. Fusion 4 sequence is shown in purple. Structures are from Brantl and Wagner (3) (A), Lucks et al. (4) (B, C) and Takahashi et al. (5) (D, E).

Supplementary Table S5: Averages and standard deviations (MEFL) plotted in Figure 4B and 4C. Values have been rounded to the nearest integer for ease of interpretation.

	No antisense (average)	With antisense (average)	No antisense (SD)	With antisense (SD)
Transcriptional WT	38699	5849	4207	1132
Dual control WT	94590	3671	9334	455
Transcriptional Mutant 1	38311	7369	4557	776
Dual control Mutant 1	81071	2832	14435	1116
Transcriptional Mutant 2	37943	14133	2518	867
Dual control Mutant 2	78197	17278	4814	3053
Transcriptional Fusion 3	36984	6935	3957	844
Dual control Fusion 3	87808	2675	8282	432
Transcriptional Fusion 4	48829	10917	4764	2190
Dual control Fusion 4	138298	5338	9637	1056

Supplementary Table S6: Standard deviations for the percent repressions presented in Figure 5B with full-length antisense. Bolded cells indicate the diagonal.

	WT full AS	Mut 1 full AS	Mut 2 full AS	Fus 3 full AS	Fus 4 full AS
WT sense	**0.77**	11.31	11.82	20.47	14.14
Mut 1 sense	9.89	**1.23**	12.25	20.17	22.70
Mut 2 sense	13.39	12.32	**5.50**	25.33	19.28
Fus 3 sense	16.57	33.62	24.14	**3.56**	20.95
Fus 4 sense	10.78	14.73	13.43	11.11	**3.78**

Supplementary Table S7: Standard deviations for the percent repression presented in Figure 5C with truncated antisense. Bolded cells indicate the diagonal.

	WT trunc AS	Mut 1 trunc AS	Mut 2 trunc AS	Fus 3 trunc AS	Fus 4 trunc AS
WT sense	**0.62**	11.42	10.99	19.93	18.07
Mut 1 sense	15.76	**1.51**	17.57	21.57	16.72
Mut 2 sense	8.61	10.06	**4.13**	17.50	7.83
Fus 3 sense	12.92	9.30	12.55	**0.57**	10.65
Fus 4 sense	17.02	12.14	16.28	13.62	**0.81**

References

1. Bustin, S.A., Benes, V., Garson, J.A., Hellemans, J., Huggett, J., Kubista, M., Mueller, R., Nolan, T., Pfaffl, M.W., Shipley, G.L., et al. (2009) The MIQE Guidelines: minimum information for publication of quantitative real-time PCR experiments. *Clin. Chem.*, **55**, 611-622.

2. Chappell,J., Takahashi,M.K. and Lucks,J.B. (2015) Creating small transcription activating RNAs. *Nat. Chem. Biol.*, **11**, 214–220.

3. Brantl,S. and Wagner,E.G. (2000) Antisense RNA-mediated transcriptional attenuation: an in vitro study of plasmid pT181. *Mol. Microbiol.*, **35**, 1469–1482.

4. Lucks,J.B., Qi,L., Mutalik,V.K., Wang,D. and Arkin,A.P. (2011) Versatile RNA-sensing transcriptional regulators for engineering genetic networks. *Proc. Natl. Acad. Sci. U.S.A.*, **108**, 8617–8622.

5. Takahashi,M.K. and Lucks,J.B. (2013) A modular strategy for engineering orthogonal chimeric RNA transcription regulators. *Nucleic Acids Research*, **41**, 7577–7588.
